# ADFireNet: An Anchor-Free Smoke and Fire Detection Network Based on Deformable Convolution

**DOI:** 10.3390/s23167086

**Published:** 2023-08-10

**Authors:** Bin Li, Peng Liu

**Affiliations:** 1 School of Computer Science, Northeast Electric Power University, Jilin 132011, China; 2 Gongqing Institute of Science and Technology, No. 1 Gongqing Road, Gongqing 332020, China

**Keywords:** smoke and fire detection, deformable convolution, anchor-free detection network

## Abstract

In this paper, we propose an anchor-free smoke and fire detection network, ADFireNet, based on deformable convolution. The proposed ADFireNet network is composed of three parts: The backbone network is responsible for feature extraction of input images, which is composed of ResNet added to deformable convolution. The neck network, which is responsible for multi-scale detection, is composed of the feature pyramid network. The head network outputs results and adopts pseudo intersection over union combined with anchor-free network structure. The head network consists of two full convolutional subnetworks: the first is the classification sub-network, which outputs a classification confidence score, and the second is the regression sub-network, which predicts the parameters of bounding boxes. The deformable convolution (DCN) added to the backbone network enhances the shape feature extraction capability for fire and smoke, and the pseudo intersection over union (pseudo-IoU) added to the head network solves the label assignment problem that exists in anchor-free object detection networks. The proposed ADFireNet is evaluated using the fire smoke dataset. The experimental results show that ADFireNet has higher accuracy and faster detection speeds compared with other methods. Ablation studies have demonstrated the effectiveness of DCN and pseudo IoU.

## 1. Introduction

Fire can cause serious casualties and loss of property. It is important to be able to automatically detect flames or smoke and sound alarm at the earliest opportunity. Previous fire detection methods based on physical sensors have limitations, including limited use space, high costs, and false positives. Increasing attention has been paid to fire detection algorithms based on images due to their intuitive and real-time characteristics [1,2].

Fire detection is a specific application of object detection algorithms, and many fire detection algorithms are derived from object detection algorithms [3,4]. Object detection networks can be roughly divided into two categories: two-stage and one-stage object detection networks. In the first stage, two-stage detection networks first generate many anchors based on rules preset by the region proposal network (RPN), then retains a certain number of anchors after non-maximum suppression (NMS). In the second stage, two-stage detection networks regress the instances anchors based on the ground truth boxes. Fire detection methods based on these algorithms include that described by Zeng et al. [5], which is based on Faster Region-based Convolutional Neural Network (R-CNN) [6]. The original feature extraction network is replaced by Inception ResNet V2, which achieves better results in open scenes. Yu et al. [7] improved Mask R-CNN [8], introduced a bottom-up feature fusion structure in Feature Pyramid Network (FPN) [9], and improved the loss function to make fire position detection more accurate. Barmpoutis et al. [10] used Faster R-CNN to obtain the rectangular fire region and then projected it to the Glassman manifold using the linear dynamic algorithm so that the network could reduce the false positive rate of fire with similar color regions. However, the two-stage object detection networks have complex structures, which makes training more difficult and impacts detection speed. To improve the above shortcomings, one-stage object detection algorithms were proposed.

The representative of one-stage algorithms is the YOLO series algorithms, which do not need RPN but directly obtain the final bounding boxes from the input image. Jiao et al. [11] used the YOLOv3 [12] algorithm for forest fire detection. Benefiting from the simpler network structure of one-stage detection algorithms, the whole network can be deployed on an Unmanned Aerial Vehicle (UAV) for forest patrol. Yue et al. [13] added the K-means++ clustering algorithm to YOLOv3 to further select the generated anchors, which reduced the training loss caused by the mismatching of anchors and ground truths and increased calculation compared with the original YOLOv3 algorithm. Qin et al. [14] proposed a fire detection algorithm combining the classification model in deep learning with the object detection model. First, the fire image is classified by depth-wise separable convolution, and information on the fire position is then outputted using a regression function in YOLOv3, which avoids the loss of accuracy caused by using YOLOv3 simultaneously for classification and location. Compared with two-stage algorithms, one-stage fire detection algorithms simplify network structures to a certain extent, although they still do not completely remove the influence of anchors.

Anchors lead to many parameters in the whole network; this affects the detection speed regardless of whether the networks are one-stage or two-stage detection networks. Second, the anchors are generated in advance by artificially preset parameters, thus the parameter settings have a great impact on the detection results, affecting the generalization ability of the networks. Finally, anchors of fixed size make it difficult to accurately enclose irregular shapes such as fire and smoke.

Anchor-free object detection algorithms were introduced after the publication of RetinaNet [15] and full convolutional network (FCN) [16]. The structure of the anchor-free object detection algorithm resembles that of RetinaNet: the head network uses a full convolutional structure, the parameters of predicted boxes are directly obtained by regression of key points, and the representative algorithms include FCOS [17] and CenterNet [18]. Anchor-free object detection algorithms eliminate anchors and have fewer parameters and faster detection speeds; these enable them to meet the real-time requirements of fire detection tasks.

In anchor-based networks, multiple anchors are generated at each pixel using preset parameters; these anchors are assigned as positive or negative samples based on the IoU threshold. After that, a certain proportion of positive and negative samples are transmitted to the network for subsequent training. Therefore, the process of generating anchors also involves label assignment. Some studies have also proved that selecting a better assignment ratio can improve the training effect of networks. However, in common anchor-free networks, all points in the training area directly regress the box without further assignment, leading to some points that are far from the instances’ center or background also participating in the training process, which has certain adverse effects on training.

In recent years, some anchor-free object detection networks have also realized this problem and tried to resolve it. For example, FCOS [17] added a CenterNess branch in the head network parallel with the classification and regression branches. First, all points falling in the ground truth boxes are regarded as positive samples. The CenterNess is then used to calculate the weight of the output results to reduce the influence of some false positive points. Feature selective anchor-free (FSAF) [19] was combined with the anchor-based and anchor-free network structures. However, all these methods need to introduce extra parameters or add new network structures, increasing network complexity. In summary, all the above methods are based on the same idea: points near the center of the object tend to produce higher-quality bounding boxes. Based on this idea, pseudo-IoU [20] is used in this paper to solve the label assignment problem in the anchor-free fire detection network. After each layer of the output of FPN, points can be mapped back to the position of the original input feature map. Previous anchor-free object detection networks will regard all points falling into the ground truth box as positive samples, but the pseudo-IoU will further assign these points for later stages.

Previous fire detection networks also have another defect: they ignore the complexity of the shapes of fire and smoke instances, and the common convolution used in the previous methods inevitably leads to a lack of shape feature sampling ability when facing such complex shapes. However, deformable convolution (DCN) [21] can make the sampling points in the convolution process shift with the shape of the instances, making it more suitable for sampling fire and smoke.

To ensure real-time performance and strengthen the sampling capability of complex shapes, we proposed an anchor-free fire and smoke detection network based on DCN and pseudo-IoU called ADFireNet. The network uses a three-stage structure like previous anchor-free networks. The DCN in the backbone network has a stronger feature extraction ability for irregular fire and smoke. Adding pseudo-IoU after FPN solves label assignment problems in anchor-free networks. ADFireNet has many potential application scenarios, for example, using ADFireNet to monitor forest fires or for early detection and alarm of smoke and flames in urban buildings.

This paper makes the following contributions:

(1) Compared with traditional convolution, DCN is more suitable for extracting features of complex instance shapes such as fires and smoke.
(2) The simple metric, pseudo-IoU, is added to improve the problem of poor training caused by a lack of label assignment in common anchor-free detection networks, and there is no need to add an additional network structure.
(3) Compared with other fire detection algorithms, ADFireNet has fewer parameters and enhances the sampling ability of complex shapes, which can meet the real-time detection requirements and also has high accuracy.

This paper is structured as follows. In Section 2, the related DCN and pseudo-IoU are introduced. In Section 3, the detailed structure of the ADFireNet is introduced. In Section 4, we evaluate ADFireNet using the fire and smoke dataset and analyze the experimental results. We summarize the study in Section 5.

## 2. Related Work

### 2.1. Deformable Convolution Network

**Figure 1:**
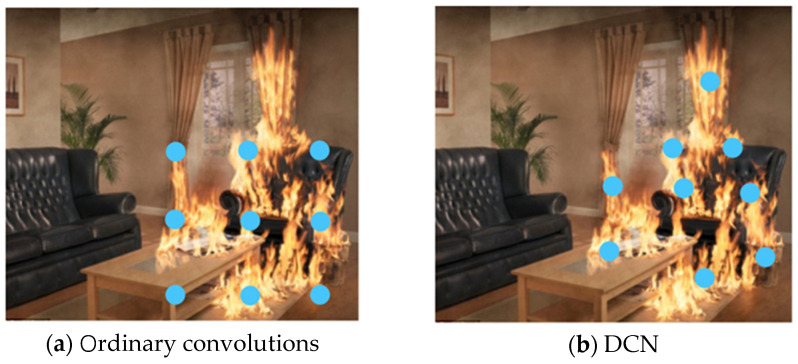
Schematic of sampling points of traditional convolution and DCN.

In traditional convolution, the sampling points are evenly and regularly distributed. For example, in Figure 1a, the sampling points in the ordinary 3 × 3 convolution are regular. However, for instances with irregular shapes like fire and smoke, we hope that the sampling points of the convolution kernel will also be uneven and irregular to better fit the contour of instances. In other words, the convolution can have a “deformation” ability, as shown in Figure 1b. Therefore, we use DCN in the ADFireNet network backbone to adapt to the irregular shapes of flames and smoke.

**Figure 2:**
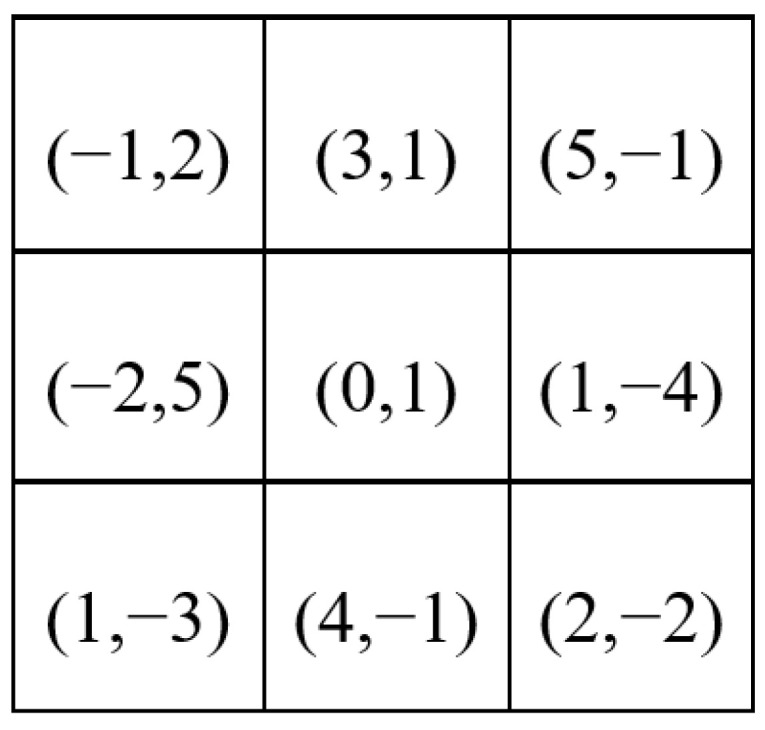
Offsets generated by DCN.

The sampling point positions of the DCN can be offset, learned through the 4-layer convolution. For the input feature map with size H × W × C (H, W, and C represent the height, width, and channels of the feature map, respectively), a convolution kernel with a 3 × 3 size is used for sampling in the next step; that is, there are a total of nine sampling points in the convolution kernel. Before this step, the feature map was first made to pass through the 4-layers convolution kernels in the DCN branch, and the paddings of all convolution kernels were set to “same” to keep the feature map size unchanged. The last convolution kernel is set as 3 × 3 × (2 × 9), hence the offset output with size H × W × (2 × 9) is obtained. H × W means that each point on the input feature map has a corresponding offset. (2 × 9) indicates that there are offsets in the X and Y axes for all nine sampling points. We assume that the offsets can be expressed as shown in Figure 2 after combining the X and Y axes, and the coordinates in each grid represent the offset distance of the sampling points.

For the next convolution step, the H × W pixels on the feature map are sampled in a 3 × 3 kernel using a sliding window. The positions of the nine sampling points add their offsets, leading to the sampling of positions that are uneven and irregular, i.e., the sampling point locations deviate. After training, the locations of sampling points can better fit the shape of fire or smoke instances, as shown in Figure 1b. As the offsets are typically fractional, they need to be implemented via bilinear interpolation to find the nearest integer coordinates.

### 2.2. Label Assignment and Pseudo-IoU

In anchor-free object detection networks, the parameters of the predicted box are directly regressed from points, thus boxes generated by points near the center of instances are usually of higher quality. Based on this idea, pseudo-IoU [20] is used to solve the label assignment problem in ADFireNet. After passing through FPN, points can be mapped back to their positions on the original input feature map. In previous networks, points falling into the ground truth box were regarded as positive samples but the pseudo-IoU further calculates these points for label assignment.

For point P obtained after mapping back, the distances between P and the four edges of ground truth box *T* can be denoted as t*, b*, l* and r*, and the area of *T* can be denoted as S_T_ and calculated as:

(1) $$S_{T}=(t^{*}+b^{*})\times (l^{*}+r^{*})$$

Based on the *T* parameters, a pseudo box, *F*, centered on point *P* and with the same area as *S_T_* can be established. The distances between point *P* and the four edges of *F* are determined as:

(2) $$t_{F}=d_{F}=(t^{*}+b^{*})/2$$

(3) $$l_{F}=r_{F}=(l^{*}+r^{*})/2$$

The parameters of part *I*, which intersects with *T* and *F* are defined as:

(4) $$t_{I}=\min\left(t^{*},t_{F}\right)\;b_{I}=min(b^{*},b_{F})$$

(5) $$l_{I}=\min\left(l^{*},l_{F}\right)\;r_{I}=min(r^{*},r_{F})$$

The IoU of the ground truth box *T* and pseudo box *F* is calculated as:

(6) $$IoU=\frac{\left|A\cap B\right|}{\left|A\cup B\right|}=\frac{S_{I}}{S_{T}+S_{F}-S_{I}}$$

The IoU calculated in this way is derived from the ground truth box *T* and the pseudo box *F*, and hence is called the pseudo-IoU. After the pseudo-IoU is calculated, it is compared against the threshold of 0.5. If the value is greater than 0.5, this point is set as a positive sample, otherwise, it is set as a negative sample. After the pseudo-IoU is added for label assignment, points that were originally regarded as positive samples but were far from the instance center are re-assigned as negative samples. Before reassignment, these points generate low-quality bounding boxes. However, after reassignment, these bounding boxes can be removed to improve the training effect of the network.

## 3. Approach

### 3.1. Architecture of ADFireNet

**Figure 3:**
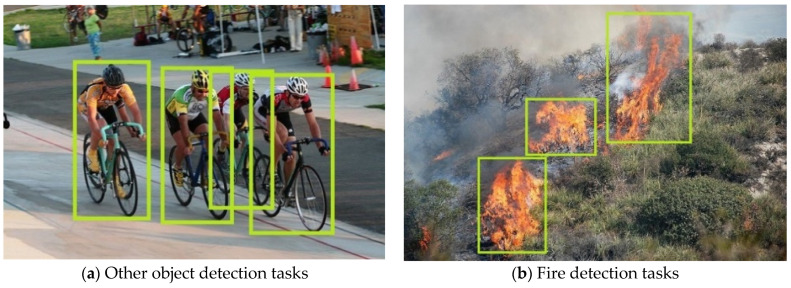
Comparison of cyclists and fire detection.

Compared with other object detection tasks, fire detection is associated with some difficulties: the size and shape of fire and smoke instances are not fixed. As shown in Figure 3a, four instances belonging to the same category of cyclists have similar aspect ratios and sizes of bounding boxes; however, fire detection does not have these characteristics. As shown in Figure 3b, the sizes and aspect ratios of the three fire instances are different. Therefore, the anchor-free network is used in this paper. In anchor-based networks, the size and aspect ratio of anchors are artificially set in advance, which is not suitable for tasks such as fire detection where the size and shape of the instances are variable. Fire detection has the above characteristics because the fire and smoke instances in the detection task are not rigid bodies but complex and variable. DCN is used to solve the feature extraction problem of fire and smoke shapes, and FPN combined with pseudo-IoU is used to solve the problem of changing sizes.

**Figure 4:**
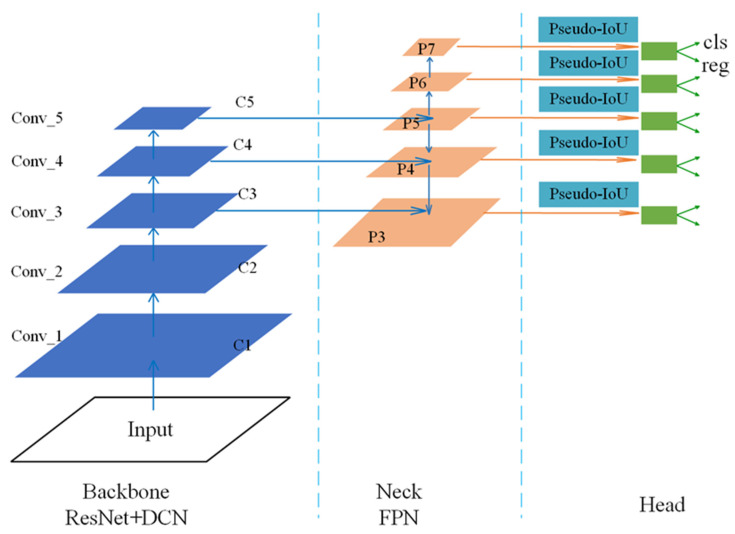
The structure of ADFireNet.

ADFireNet is divided into three parts: The backbone network responsible for feature extraction of input images, which is composed of ResNet [22] combined with DCN. The neck network, which is responsible for multi-scale detection, is composed of FPN. The head network consists of two full convolutional sub-networks: the first is the classification sub-network (cls), which outputs the classification confidence score, and the second is the regression sub-network (reg), which predicts the parameters of bounding boxes. The ADFireNet structure is shown in Figure 4.

**Figure 5:**
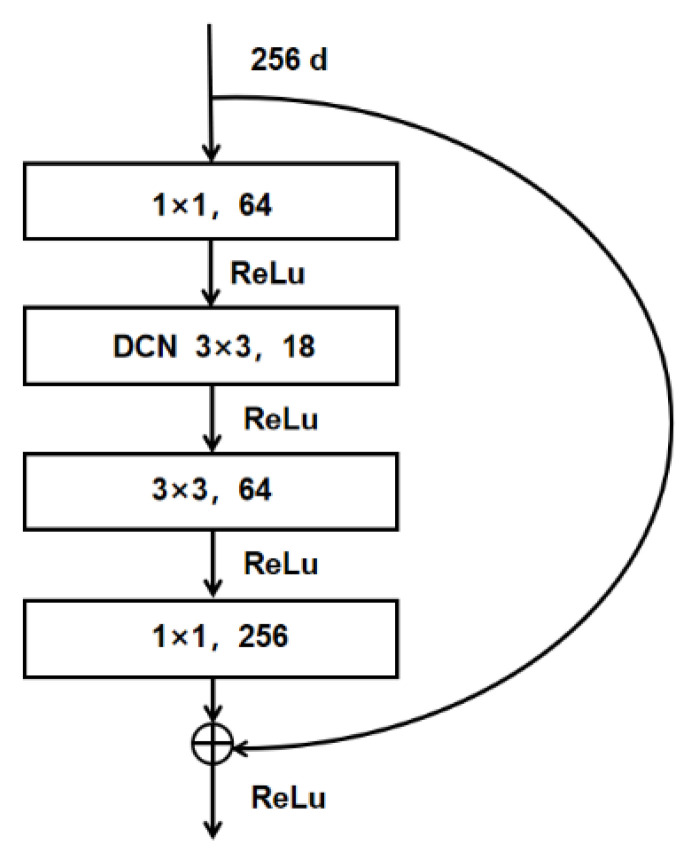
Residual block structure after adding DCN.

Considering the number of parameters and detection speed, ResNet-50 is used as a backbone network in ADFireNet. Resnet-50 is composed of five convolution blocks: conv_1–conv_5. The five feature maps generated by these five convolution blocks are denoted as C1–C5 in Figure 4. The down-sampling steps between each convolution block increase exponentially by 2, such that the down-sampling rate of the Ci layer is 2i. Each convolution block consists of several residual blocks. To avoid adding too much computation, DCN is added to the last residual block of the three convolution blocks: conv_3, conv_4, and conv_5. The shape features of the flame extracted by adding the residual block of DCN are transmitted to the P3, P4, and P5 layers of the subsequent FPN. The residual block structure obtained after adding DCN is shown in Figure 5.

The neck network of ADFireNet is a feature pyramid network (FPN). As shown in Figure 4, the neck network has five layers—P3, P4, P5, P6, and P7—that are used to detect targets of different sizes. Among them, P3, P4, and P5 are directly generated by combining C3, C4, and C5 of the backbone network with the top-down structure, while P6 and P7 are generated by P5 with the down-sampling step of 2 to detect larger size instances. In FPN, the number of sampling steps increases exponentially from bottom to top, and the number of steps in the Pi layer is 2i. For example, the sampling steps in layer P3 is 8 and that in layer P7 is 128. The higher the number of layers, the greater the number of sampling steps, and the smaller the size of the output feature map, and vice versa.

In the anchor-based target detection network, the FPN layer used for detection is specified based on the size of the anchor box, while in the anchor-free network, the FPN layer used for detection is determined by the target size range. The target size is obtained from the ground truth in the labeled image. The ground truth size consists of four parameters represented by distances from a target point to the four sides of the ground truth box. The distances from a point in the target to the upper, lower, left, and right edges of the ground truth box are marked as t*, b*, l*, and r*, respectively. Assuming that Mi is the upper limit of the target size of layer Pi in the FPN, if a point in layer Pi meets $M_{i}\leq max(t^{*},b^{*},r^{*})<M$ and the point is assigned a positive label by pseudo-IoU, the point will be transmitted to the subsequent detection head network to obtain the detection box parameters. Otherwise, the Pi layer ignores this point, which is processed by other layers of the FPN. We set the image size of the fire dataset used in the experiment to 640 × 640 pixels, thus M3–M7 are set to 64,128,256,512 and ∞, respectively. In this way, targets of different sizes are assigned to each layer of FPN for detection. The lower layer of FPN is responsible for detecting large targets and the upper layer is responsible for detecting small targets.

After extracting features through the backbone network and FPN, the pseudo-IoU assigns positive or negative labels to points P3–P7 in the feature maps. The location of any point on the feature map of the Pi layer can be mapped back to the original input image. If the mapping coordinates of the point fall into the ground truth box in the original image, this point is designated as a positive sample, otherwise, the point is designated as a negative sample.

**Figure 6:**
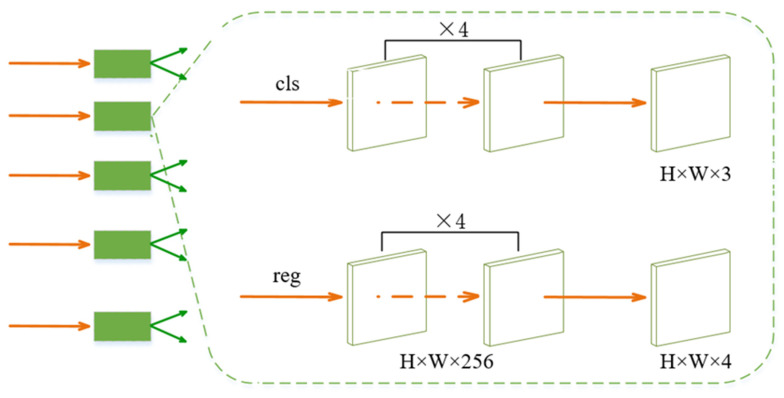
The structure of classification and regression sub-networks.

The head network is connected to the FPN in each layer and is composed of classification and regression sub-networks. It outputs the classification confidence score and the parameters of the bounding box, respectively. The classification network is composed of four layers of 3 × 3 × 256 full convolutional network. A 3-channel convolution layer is connected to them to divide each point into three categories: fire, smoke, and background. The first four layers of the regression sub-network are similar to those of the classification sub-network, except that the number of channels in the last layer is four, representing the distances from this point to the four edges of the predicted bounding box. For each point in the input image, the network gives the category confidence score and the size of the bounding box. The head network structure is shown in Figure 6:

### 3.2. Loss Function

Corresponding to the classification and regression sub-network of the head network, the loss function is composed of two parts: the focal loss [15] $L_{fl}$ used for classification and IoU loss [23] L_iou_ used for regression. The loss function is defined as follows:

(7) $$L\left(A_{\left(x,y\right)},B_{\left(x,y\right)}\right)=\frac{1}{N_{pos}}\sum_{\left(x,y\right)}L_{fl}\left(C_{A_{x,y}},C_{B_{x,y}}\right)+\frac{\lambda }{N_{pos}}\sum_{\left(x,y\right)}L_{iou}\left(R_{A_{x,y}},R_{B_{x,y}}\right)$$

$A_{\left(x,y\right)}$ represents all the training samples and $B_{x,y}$ represents all the ground truth boxes. $C_{A_{x,y}}$ and $R_{A_{x,y}}$ represent the outputs of the classification and regression sub-networks. $C_{B_{x,y}}$ and $R_{B_{x,y}}$ represent the labels of ground truth. $N_{pos}$ is the number of selected positive samples; this parameter is affected by pseudo-IoU. λ is the balance weight for IoU loss and is set to 1.0.

## 4. Experimental Section

### 4.1. Experimental Dataset

**Figure 7:**
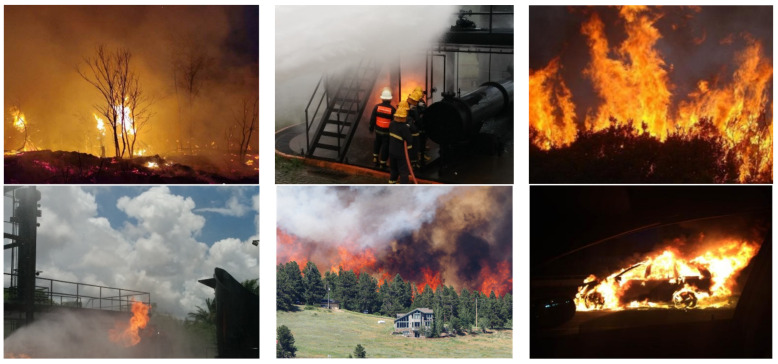
Sample images from the experimental dataset.

To test the performance of the ADFireNet network, an experimental dataset was established using the FireDunning dataset and fire images collected from the Internet. The experimental dataset used in this study consists of 5322 images from the FireDunning dataset, as well as 2000 fire and smoke images collected from the Internet. The 7322 pictures in the experimental dataset used in this paper include fire and smoke images from warehouses, forests, road vehicles, and other scenes. We labeled fire and smoke images in all scenes. All images are labeled in VOC dataset format, with each flame or smoke instance containing category information (fire, smoke, and background) and coordinates of the upper left and lower right corners where the bounding box is located. In subsequent experiments, the size of the input image was set to 640 × 640 and all images were divided into training and testing sets in a 4:1 ratio. Figure 7 shows several images from the experimental dataset.

### 4.2. Implementation Details

During network training, the Adam optimizer was used to update the weights, epochs were set to 200, momentum was set to 0.9, weight decay was set to 0.0005, batch size was set to 128, and initial learning rate was set to 0.0001. The operating system used was Ubuntu 18.04LTS. Python version 3.7, PyTorch version 1.7.0, CUDA version 11.0, and NVIDIA GeForce 3060 GPU were also used.

### 4.3. Evaluation Methodology

We used two criteria to evaluate flame and smoke detection algorithms. One criterion is mAP (mean Average Precision) of 0.5, which is commonly used in object detection algorithms; that is, if the IoU between the bounding box given by the network and the ground truth box is greater than 0.5, the detection is judged to be a success, otherwise, the detection is a failure. The other criterion is frames per second (FPS), which refers to the number of images that can be detected per second and is used to evaluate the detection speed of the network.

### 4.4. Flame and Smoke Detection Results

The experiment compared ADFireNet with a representative anchor-based detection algorithm, Faster-RCNN [10], and an anchor-free algorithm, FCOS [17], which adopt ResNet-50 [22] and ResNet-101 [22] as backbone networks, respectively. The experimental results are shown in Table 1.

The detection results show that:

(1) The anchor-based method, Faster-RCNN, lags behind anchor-free methods in terms of detection speed. It also cannot meet real-time requirements and has worse performance in terms of accuracy.
(2) The FCOS and ADFireNet networks are both anchor-free frame networks. The ADFireNet network does not have a center-less branch, and the classification network adopts a single-branch multi-classification method while the FCOS network adopts a multi-branch parallel binary classification method. Therefore, its detection speed is faster than that of the FCOS network, and its detection accuracy, mAP, is 2.3% better than that of the FCOS network.
(3) The number of layers in the backbone network has a significant impact on the overall performance of the network. When the backbone network is changed from ResNet-101 to ResNet-50, the mAP of ADFireNet decreases by 1.8%, but the FPS value increases by 8%. Generally speaking, using a backbone network with fewer layers can improve detection speed, but reducing the number of layers can decrease the ability to extract features and affect detection accuracy. Therefore, the backbone network can be selected based on the actual requirement of the detection task.

### 4.5. Ablation Studies

To verify the effectiveness of pseudo-IoU and DCN in ADFireNet, we conducted ablation studies on pseudo-IoU and DCN, respectively. Table 2 shows the results of the ablation study. In the ablation study, we used ADFireNet_101 and ADFireNet_50 as baselines. Table 2 lists the mAP results of four ablation networks: networks that use ResNet-101 as the backbone and do not use DCN (indicated as Without DCN_101 layers in Table 2), networks that use ResNet-50 as the backbone and do not use DCN (indicated as Without DCN_50 layers in Table 2), networks that use ResNet-101 as the network and do not use pseudo-IoU (indicated as Without pseudo-IoU_101 layers), and networks that use ResNet-50 as the backbone and do not use pseudo-IoU (indicated as Without pseudo-IoU_50 layers).

The mAP values of ADFireNet_101 and ADFireNet_50 are higher than those of corresponding networks without DCN or pseudo IoU. The results of the isolation experiment demonstrate the effectiveness of pseudo-IoU and DCN in ADFireNet.

**Figure 8:**
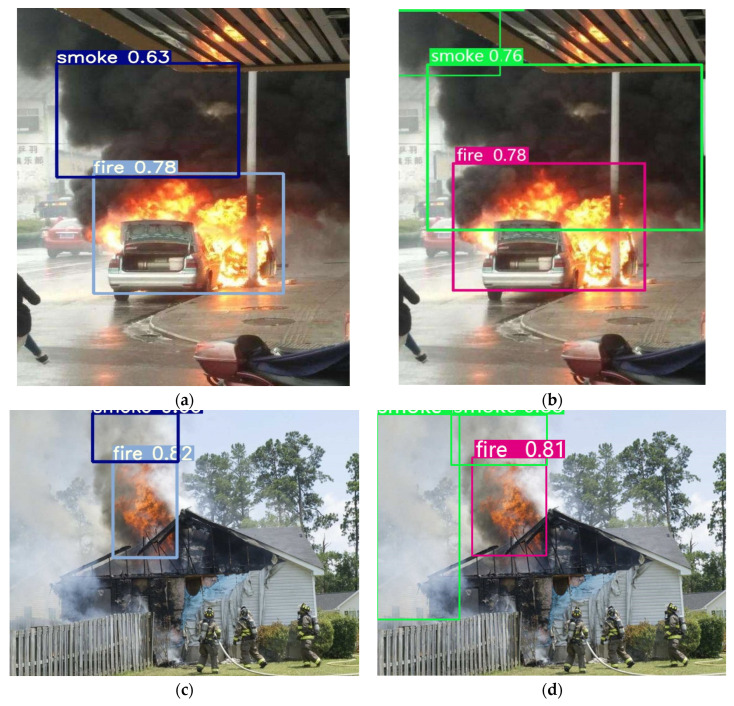
The impact of DCN on the detection performance of ADFireNet.

Figure 8 visually illustrates the impact of DCN on detection performance. Figure 8a shows the effect of using the without DCN_50 layers for flame and smoke detection. ADFireNet_50 is used to detect the same scene, as shown in Figure 8b. Figure 8c shows the effect of using the without DCN_50 layers for flame and smoke detection in another scene. ADFireNet_50 is used to detect the same scene, as shown in Figure 8d. DCN has a greater ability to extract features of irregular targets, for example, flames and smoke. When using AFFireNet_50 with the help of DCN to detect the first scene, the range of smoke contained in the detection box is larger, the positioning of smoke is more accurate, and the smoke in the upper left corner is detected. In the second scenario, AFFireNet_50 detected white smoke that had not been detected before on the left side. The detection effect in Figure 8 demonstrates that adding DCN can improve the positioning accuracy of the network and detect previously missed instances.

### 4.6. Visual Effects

**Figure 9:**
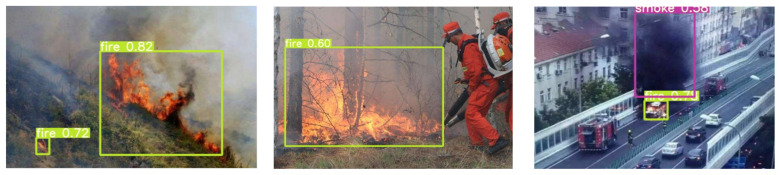

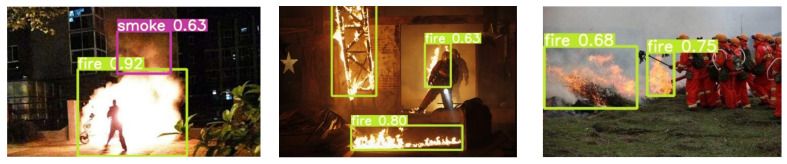
Part of the detection results pictures.

Part of the detection results pictures are shown in Figure 9. The results show that the proposed AAA accurately detects smoke and fire in both forest and urban scenes.

## 5. Conclusions

In this paper, we proposed an anchor-free fire detection network termed ADFireNet. The deformable convolution network added to the backbone network can better extract the complex shape features of fire and smoke, and the pseudo-IoU added to the head network can further assign points to obtain better training effects. The experimental results showed that, compared with other methods, the ADFireNet network has higher detection accuracy, fewer parameters, and faster detection speeds.

## Figures and Tables

**Table 1:** Flame and smoke detection results.

Method	Backbone	mAP 0.5 (%)	FPS
Faster-RCNN	ResNet-101	78.6	12
FCOS_101	ResNet-101	83.1	17
FCOS_50	ResNet-50	80.0	39
ADFireNet_101(ours)	ResNet-101	85.4	24
ADFireNet_50 (ours)	ResNet-50	83.6	32

**Table 2:** Ablation Study results.

Method	mAP 0.5 (%)
Without DCN_101 layers	83.1
Without DCN_50 layers	79.3
Without pseudo-IoU_101 layers	83.4
Without pseudo-IoU_50 layers	80.5
ADFireNet_101	85.4
ADFireNet_50	83.6

## Data Availability

Not applicable.

## References

[1] Yang Z., Bu L., Wang T., Yuan P., Jineng O. (2020). Indoor Video Flame Detection Based on Lightweight Convolutional Neural Network. Pattern Recognit. Image Anal..

[2] Ye S., Bai Z., Chen H., Bohush R., Ablameyko S. (2017). An effective algorithm to detect both smoke and flame using color and wavelet analysis. Pattern Recognit. Image Anal..

[3] Yuan J., Wang L., Wu P., Gao C., Sun L. (2018). Detection of wildfires along transmission lines using deep time and space features. Pattern Recognit. Image Anal..

[4] Xiong S., Li B., Zhu S. (2023). DCGNN: A single–stage 3D object detection network based on density clustering and graph neural network. Complex Intell. Syst..

[5] Zeng J., Lin Z., Qi C., Zhao X., Wang F. An improved object detection method based on deep convolution neural network for smoke detection. Proceedings of the 2018 International Conference on Machine Learning and Cybernetics (ICMLC).

[6] Ren S., He K., Girshick R., Sun J. Faster r–cnn: Towards real–time object detection with region proposal networks. Proceedings of the 29th Annual Conference on Neural Information Processing Systems.

[7] Yu L., Liu J. (2020). Flame image recognition algorithm based on improved mask R–CNN. Comput. Eng. Appl..

[8] He K., Gkioxari G., Dollár P., Girshick R. Mask r–cnn. Proceedings of the Proceedings of the IEEE International Conference on Computer Vision.

[9] Li B., Lu Y., Pang W., Xu H. (2023). Image Colorization using CycleGAN with semantic and spatial rationality. Multimed. Tools Appl..

[10] Barmpoutis P., Dimitropoulos K., Kaza K., Nikos G. Fire Detection from Images using Faster R–CNN and Multidimensional Texture Analysis. Proceedings of the ICASSP 2019—2019 IEEE International Conference on Acoustics, Speech and Signal Processing (ICASSP).

[11] Jiao Z., Zhang Y., Xin J., Mu L., Yi Y., Liu H., Liu A. Deep learning based forest fire detection approach using UAV and YOLOv3. Proceedings of the 2019 1st International Conference on Industrial Artificial Intelligence (IAI).

[12] Redmon J., Farhadi A. (2018). Yolov3: An incremental improvement. arXiv.

[13] Yue C., Ye J. Research on Improved YOLOv3 Fire Detection Based on Enlarged Feature Map Resolution and Cluster Analysis. Proceedings of the International Conference on Computer Big Data and Artificial Intelligence (ICCBDAI 2020).

[14] Qin Y.-Y., Cao J.-T., Ji X.-F. (2021). Fire detection method based on depthwise separable convolution and yolov3. Int. J. Autom. Comput..

[15] Lin T.-Y., Goyal P., Girshick R., He K., Dollár P. Focal loss for dense object detection. Proceedings of the IEEE International Conference on Computer Vision.

[16] Long J., Shelhamer E., Darrell T. Fully convolutional networks for semantic segmentation. Proceedings of the IEEE Conference on Computer Vision and Pattern Recognition.

[17] Tian Z., Shen C., Chen H., He T. (2020). Fcos: A simple and strong anchor–free object detector. IEEE Trans. Pattern Anal. Mach. Intell..

[18] Law H., Deng J. Cornernet: Detecting objects as paired keypoints. Proceedings of the European Conference on Computer Vision (ECCV).

[19] Zhu C., He Y., Savvides M. Feature selective anchor–free module for single–shot object detection. Proceedings of the IEEE/CVF Conference on Computer Vision and Pattern Recognition, Long Beach Convention & Entertainment Center.

[20] Li J., Cheng B., Feris R., Xiong J., Huang T.S., Hwu W.-M., Shi H. Pseudo–iou: Improving label assignment in anchor–free object detection. Proceedings of the IEEE/CVF Conference on Computer Vision and Pattern Recognition Workshops (CVPRW).

[21] Dai J., Qi H., Xiong Y., Li Y., Zhang G., Hu H., Wei Y. Deformable convolutional networks. Proceedings of the IEEE International Conference on Computer Vision.

[22] He K., Zhang X., Ren S., Sun J. Deep residual learning for image recognition. Proceedings of the IEEE Conference on Computer Vision and Pattern Recognition.

[23] Yu J., Jiang Y., Wang Z., Cao Z., Huang T. UnitBox: An Advanced Object Detection Network. Proceedings of the 24th ACM International Conference on Multimedia.

